# Dimethyladenosine Transferase 1 Homolog Promotes Human Gastric Carcinoma Cell Proliferation and Inhibits Apoptosis via the *AKT* Pathway

**DOI:** 10.5152/tjg.2023.22169

**Published:** 2023-08-01

**Authors:** Guang-yi Liu, Huan Wang, Rui Ran, Yi-cheng Wang, Yang Li

**Affiliations:** 1 Department of Gastrointestinal Surgery, The Second Affiliated Hospital of Chongqing Medical University, Chongqing, China; 2 Department of Health Management Center, The Second Affiliated Hospital of Chongqing Medical University, Chongqing, China

**Keywords:** *DIMT1*, gastric carcinoma, proliferation, apoptosis, gene regulation

## Abstract

**Background/Aims:**

Our previous work identified the dimethyladenosine transferase 1 homolog as a novel prognostic factor for detecting human gastric carcinoma with high sensitivity and specificity. The high expression of dimethyladenosine transferase 1 is closely associated with the occurrence and progression of gastric carcinoma. However, the underlying mechanism of dimethyladenosine transferase 1 for the occurrence and development of gastric carcinoma is not well elucidated yet.

**Materials and Methods:**

In our present study, the biological role of dimethyladenosine transferase 1 on cell proliferation, apoptosis, and cell cycle progression in human gastric carcinoma cells was investigated through *in vitro* and *in vivo* assays by the overexpression and knockdown of dimethyladenosine transferase 1 2-way authentication method.

**Results:**

We found that the overexpression of dimethyladenosine transferase 1 significantly promotes cell proliferation (*P* < .001) and inhibition of cell apoptosis (*P* < .01) in SGC-7901 cells. However, the *in vivo* experiment results of the knockdown dimethyladenosine transferase 1 using small interfering RNAs in the MKN-45 are just the opposite. Reverse-transcriptase polymerase chain reaction and western blotting analysis revealed that overexpressed dimethyladenosine transferase 1 in SGC-7901 cells significantly activated the *AKT* pathway compared to control cells. In contrast, we found that apoptosis genes such as *Caspase-3* and *Caspase-9* were downregulated in those cells. The xenograft nude mice model exhibited increased tumor growth (*P* < .01) and weight loss (*P* < .01), with the overexpression of dimethyladenosine transferase 1 homolog in the SGC-7901 cells. These results have been further confirmed through backward verification in dimethyladenosine transferase 1 knockdown cells.

**Conclusions:**

Taken together, our results indicated that the dimethyladenosine transferase 1 plays a crucial role in stimulating cancer cell proliferation and contributes to apoptosis resistance in human gastric carcinoma. Meanwhile, it provides a potential therapeutic target for gastric carcinoma treatment and is worthy of further studies.

**Main Points**

- In histological studies, we found that dimethyladenosine transferase 1 was an independent risk factor for poor prognosis in patients with gastric cancer, and its expression level was negatively correlated with prognosis.
- Dimethyladenosine transferase 1 can promote the proliferation of gastric cancer cells, inhibit the apoptosis of gastric cancer cells, and promote the formation of new tumor vessels in nude mice.
- Dimethyladenosine transferase 1 can regulate the expression levels of *CyclinD1*, *Caspase-3, Caspase-9*, *Bax/Bcl-2*, and other proteins by activating the *AKT* signaling pathway and regulating the proliferation and apoptosis of gastric cancer cells.

## Introduction

Gastric carcinoma (GC) is a common digestive system neoplasm, representing a significant health burden that is rapidly increasing.^1^ According to the latest statistics, GC was the second most common cancer in China^2^ and has a low overall 5-year survival rate of about 20%, which is due to late diagnosis and lack of standard therapy.^3,4^ Surgery combined with chemotherapy, immunotherapy, and other therapeutic methods are common practices for GC; however, it failed to reduce the high fatality rate for GC.^5,6^ Ultimately, poor prognosis with early recurrence and metastasis are closely related to the biological characteristics of GC. Elucidating the underlying mechanisms involved in GC tumorigenesis, proliferation, apoptosis, invasion, and metastasis is essential for the development of new effective therapeutic measures. The completion of the human genome’s draft sequence revealed that GC is a complex molecular disease, and its tumorigenesis and development is closely correlated with the changes in some definite gene, such as abnormal expression, dysfunction, and so on. The key is to identify the susceptible key genes and elucidate its role in the tumorigenesis and development, which laid the foundation for the accuracy of tumor treatment.^7^

Till now, cDNA microarray analysis is a high-throughput measurement platform that is widely applied in cancer and other disease research, and the cDNA microarray results were further analyzed by the bioinformatics method.^8^ Through these combined technologies, genome expression can be measured, and gene abnormal expression and dysfunction can be demonstrated. However, still, vital gene expression profiling in human GC is a rare event. Combining Kotaro Kiga and our previous study results indicated that the dimethyladenosine transferase 1 homolog (*DIMT1*) is one of the most upregulated genes in GC tissues compared with the normal gastric tissues.^9,10^ Dimethyladenosine transferase 1, also named *HUSSY5* or *HSA9761*, is a member of the methyltransferase superfamily, located on the long arm of human chromosome 5. Dimethyladenosine transferase 1 is mainly expressed in the nucleus and has a relative molecular mass of 35 kDa. Our previous study found that *DIMT1* was highly expressed in GC tissues, and its high expression is closely correlated with differentiation, tissue invasion, lymph node metastasis, distant metastasis, and clinical Tumor Node Metastasis (TNM) staging of GC, and it has a negative impact on the prognosis of the GC patients.^10^ However, specific mechanisms need to be elucidated and require further investigation.

In recent years, gene knockdown (small interfering RNA, siRNA) or gene overexpression (Construction of lentivirus vectors carrying alphastatin gene) technology has become a specific and powerful tool to turn off or promote the expression of target genes.^11-13^ In the present work, we used gene overexpression and siRNAs targeting human *DIMT1* to increase and reduce the expression of *DIMT1* and study the impact of these technologies on cellular proliferation and apoptosis in GC cell lines. Meanwhile, we also validated these results by inoculation in nude mice.

## Materials and Methods

### Cell Culture

Human GC cell lines, MKN-45, SGC-7901, MKN-45, BGC-823, and immortalized gastric mucosal epithelial cells (GES-1), were obtained from the Chinese Academy of Sciences. The cells were cultured in Roswell Park Memorial Institute 1640 (RPMI 1640, Gibco, Grand Island, NY, USA) with 10% fetal bovine serum (Gibco) and 100 U/mL penicillin-streptomycin at 37°C and 5% CO_2_. All cell lines were cultured according to the supplier’s instructions.

### Cell Transfection

The lentivirus were purchased from HANBIO (Shanghai, China). The lentivirus plasmids contain the target gene, green fluorescent protein (*GFP*) gene, and puromycin-resistant gene. Cells in the exponential growth phase were plated in 6-well plates at 1 × 10^6^ cells/well, grown for 8 hours, and then transfected with lentivirus (Multiplicity of Infection [MOI]= 1 : 80) for 6 hours using the LipoFiterTM agent. Control cells were transfected with empty lentivirus (empty lentivirus containing the *GFP *gene and puromycin-resistant gene). All the steps were performed following the manufacturer’s instructions. These cells were transfected 3 times with lentivirus particles. Next, we filtered the cells with transfected by using puromycin. The intensity of infection was monitored by *GFP* fluorescence, reverse-transcriptase polymerase chain reaction (RT-PCR), and Western blot analysis.

### Reverse Transcriptase Polymerase Chain Reaction

Total mRNA was extracted from different cells using the Trizol reagent (Takara, Dalian, China), cDNA was then synthesized, and RT-PCR was performed using an SYBR Green PCR kit (Takara), according to the manufacturer’s instructions. Constitutively expressed glyceraldehyde-3-phosphate dehydrogenase (*GAPDH*) gene was used as an internal control. Each sample was independently tested in triplicate with a final volume of 10 μL containing 2 μL of the cDNA template, 0.3 μL of each primer, 5 μL of SYBR Green PCR master mix, and 2.4 μL of double distilled water. Reaction conditions were as follows: 1 cycle of 95° for 30 seconds followed by 40 cycles of 95° for 20 seconds and 60° for 30 seconds. Data were analyzed with the 2^–ΔΔCT^ method.

### Immunofluorescence Staining and Confocal Microscope

Stable transfected and control GC cells were seeded into 24-well plates. After 48 hours, stable transfected and control GC cells were fixed with 4% paraformaldehyde for 30 minutes and permeablized with 0.5% Triton X-100 on the ice, and then the cells were rinsed 3 times with phosphate-buffered saline (PBS) (for 10 minutes each time) and were blocked in the blocking buffer for 1 hour. Subsequently, the cells were then incubated with the primary antibody at 4°C overnight followed by staining with Cy3-conjugated red secondary antibody and 4’, 6-diamidino-2-phenylindole (DAPI), incubated for 2 hours and 10 minutes, respectively, at room temperature. Fluorescent signals were quantified with immunofluorescence and confocal microscopy.

### 5-Ethynyl-2’-deoxyuridine (EdU) Assay

A Cell-Light EdU DNA cell proliferation assay was performed according to the manufacturer’s instruction. Log phase cells were seeded in 6-well plates, and once cells became adherent, they were fixed andwashed. After EdU incubation, cells were treated with Apollo reaction cocktail, stained with DAPI, and visualized under a fluorescent microscope.

### Colony Formation Assay

Five-hundred log-phase cells were seeded in 6-well plates, and after incubation for 2 weeks, colonies were fixed. Cells were gently washed twice with PBS for 10 minutes, fixed with methanol, and stained with Giemsa. Colonies were counted under an inverted microscope. Colonies from 3 replicate wells were counted per group, and experiments were performed in triplicate.

### Apoptosis Assay

Annexin V/propidium iodide (PI) Apoptosis Detection Kit (BestBio, Shanghai, China) was used to detect apoptotic cells. Briefly, cells were cultured for 24, 48, and 72 hours at 37° in a 5% CO_2_ atmosphere. After incubation, adherent cells were detached with trypsin. These harvested cells were resuspended in complete RPMI 1640 medium and centrifuged at 1500 rpm for 5 minutes. Next, the cells were washed twice with ice-cold PBS and then stained simultaneously with Annexin V and PI, according to the manufacturer’s instructions, for 15 minutes in the dark. Finally, the binding buffer was added to each reaction tube, and the cells were analyzed by the flow cytometry method. Apoptosis was assayed by Annexin V/PI staining following manufacturer’s instructions. Briefly, the total apoptotic cells were made up of 2 parts: early apoptosis (Annexin V^+^ and PI^–^ cells) and late apoptosis (Annexin V^+^ and PI^+^ cells). The data were analyzed by the WinMDI V2.9 software.

### Western Blot Analysis

Western blot was performed following the standard protocol. Total protein was extracted by a mixed solution of lysis buffer and protease inhibitors. An equal volume of the same concentration protein was separated by 10% sulfate polyacrylamide slab gel electrophoresis and then transferred to a polyvinylidene fluoride (PVDF) membrane. Next, PVDF membranes were blocked for 2 hours with 5% nonfat milk at room temperature. Membranes were incubated overnight at 4° with primary antibodies. Polyvinylidene fluoride membranes were washed 3 times in Tris-buffered saline Tween-20 (TBST) for 15 minutes on the incubator shaker and subsequently incubated with secondary antibodies for 2 hours at room temperature. PVDF membranes were washed 3 times in TBST for 15 minutes each time, signals were detected using the ImageJ software with BeyoECL plus detection solution.

### Tumor Xenograft Model

All animal procedures were performed according to protocols approved by Chongqing Medical University of Institutional Animal Care and Use Committee. The immune-deficient nude mice (6-week-old mices randomly divided into 3 groupswith 6 in each group) were purchased from Chongqing Medical University Laboratory Animal Center. Tumor cells (2 × 10^6^ cells/each) were inoculated subcutaneously into the flank of nude mice.^14^ Tumor volume [*V* = (*D*×*d*^2^)/2 mm^3^, where *D* is the longest diameter and *d* is the shortest diameter] and mice weight were measured every third day.^15^ All the mice were sacrificed 30 days after inoculation; partial xenograft tumors were collected, weighed, and photographed. Next, the specimens were used for hematoxylin and eosin (H&E) staining and immunohistochemistry (IHC).

### Hematoxylin and Eosin Staining and Immunohistochemistry

All the xenograft tumors were formalin fixed, paraffin embedded, and then sliced into 3 μm sections for H&E staining and IHC assays. For IHC assay, paraffin slices were dewaxed in xylene and rehydrated in gradient alcohol. After 3 rinses in PBS, slices were immersed in 3% H_2_O_2_. The antigen was retrieved by the citric acid buffer and heated in the microwave with high heat for 3 minutes and low heat for 30 seconds for 3 cycles, and it was allowed to cool naturally. Tissue slices were blocked with 10% normal goat serum at room temperature for 1 hour. Slices were incubated with the primary antibody at 4°C overnight. Leaved at room temperature for 1 hour, followed by 3 washes, and the rest of the steps were performed according to the instruction of ready-to-easy Strept Avidin-Biotin Complex (SABC) test kits. Slides were photographed using an optical microscope.

### Statistical Analysis

The statistical analysis was performed by using the Statistical Package for Social Sciences 22.0 software (IBM Corp.; Armonk, NY, USA). The Student’s *t*-test was used to determine statistical differences between treatment and control values. *P* < .05 were considered to be of statistical significance. All data are presented as the mean ± SD of 3 independent experiments.

## Results

### Dimethyladenosine Transferase 1 Expression Is Significantly Increased in Gastric Carcinoma Cell Lines

**Figure 1.**
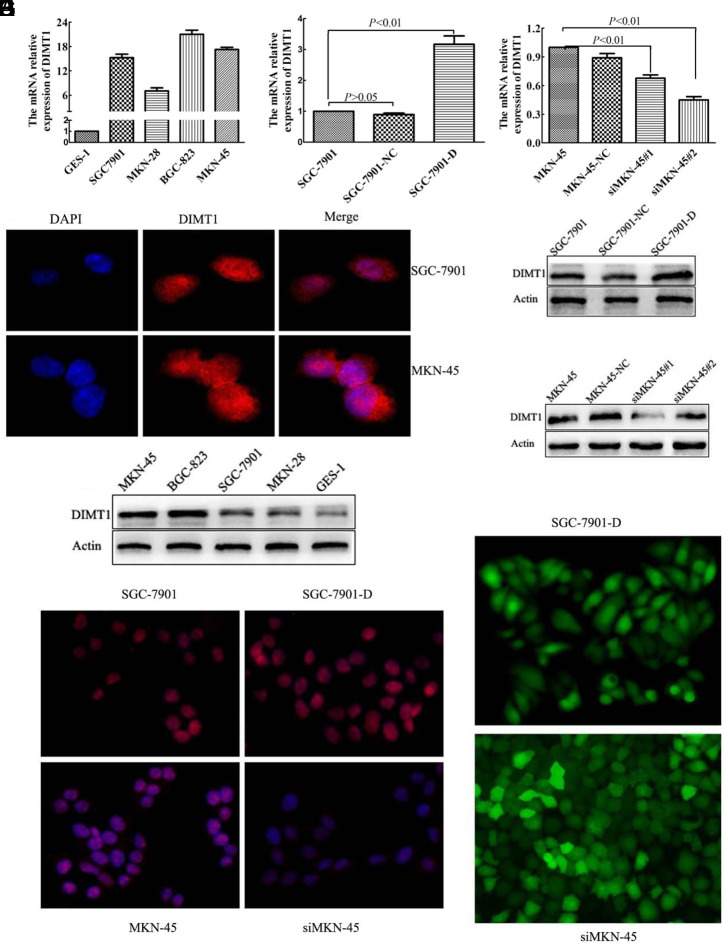
Dimethyladenosine transferase 1 (*DIMT1*) expression was upregulated in gastric carcinoma cells. (A–C) In different cell lines, the relative quantitative expression of *DIMT1* mRNA [the relative expression of gastric mucosal epithelial cells (GES-1) is set to 1 in A, SGC7901 is set to 1 in B, and MKN-45 is set to 1 in C]. (D) The fluorescence confocal images showed that *DIMT1* was localized mainly in the nucleus and a few were present in the cytoplasm; *DIMT1* was absent on the membrane. (E–G) In different gastric carcinoma cells, the protein expression of *DIMT1* had a significant difference. (H) The result of an indirect immunofluorescence assay in SGC7901, SGC-7901-D, MKN-45, and siMKN-45. (I) Successfully built-up overexpression and knockout gastric carcinoma cell line with a nearly 95% transfection rate.

In previous studies, we found that the expression of *DIMT1* in GC tissues was significantly higher compared to para-carcinoma tissues and gastric normal tissues.^10^ The high expression of *DIMT1* is positively correlated with differentiation and clinical TNM stage but negatively correlated with prognosis. Thus, we detected the expression of *DIMT1* in a series of human carcinoma cell lines (MKN-28clinical TNM st, and SGC-7901 cells) and the immortalized GES-1 cells. The quantitative RT-PCR (qRT-PCR) result suggested that the *DIMT1* expression was significantly lower in GES-1 cells, whereas its expression was higher in MKN-45 and BGC-823 cells and lower in SGC-7901 and MKN-28 cells, respectively (Figure 1A). The western blot result was the same as the qRT-PCR result (Figure 1F). The fluorescence confocal images showed that DIMT1 was localized mainly in the nucleus and a few were in the cytoplasm; it was absent on the membrane (Figure 1D). Based on these results, next, we selected SGC-7901 and MKN-45 cells to perform the overexpression and knockdown of *DIMT1*, respectively.

### Dimethyladenosine Transferase 1 Small Interfering RNA and Overexpression Significantly Changed the Protein Expression of Dimethyladenosine Transferase 1 in Gastric Carcinoma Cell Lines

Dimethyladenosine transferase 1 was knocked down and upregulated in MKN-45 cells using *DIMT1*-specific siRNA and in SGC-7901 cells using the p*EGFP*-N3-*DIMT1* vector, respectively (Figure 1I). To achieve a better silencing effect, we designed 2 siRNAs: siMKN-45#1 and siMKN-45#2. After 3 cycles of transfection, the fluorescent microscopy showed that the overexpression and interfering lentivirus transfection rate reached 95%. Next, we screened out the cells with transfected by using the puromycin with 4 times and established the stably transfected cell lines. To further verify the effect of the transfection effect, we performed qRT-PCR and western blot analysis on cell lines 48 and 72 hours, respectively, after stable transfection with the lentivirus. The mRNA level of *DIMT1* was efficiently decreased in siMKN-45 (79.5%, 71.6%; 50.4%, and 48.7%) and increased in SGC-7901-*DIMT1*-*EGFP* cells, SGC-7901-D for short (90.7% and 85.3%) following transfection with each of the *DIMT1* siRNAs and stable expression of *DIMT1*, respectively, compared with the control group (NC) and the blank group (Figure 1B and 1C). The density of western blot analysis showed that the *DIMT1* expression was decreased (47.2% and 44.3%) in siMKN-45#2 cells and (74.8% and 72.1%) in siMKN-45#1 cells compared with the MKN-45-NC cells and MKN-45 (Figure 1G), whereas the protein level of SGC-7901 cells with stable expression of *DIMT1 *increased significantly (93.2% and 88.1%; Figure 1E).

We selected siMKN-45#1 cell line (siMKN-45 is short for siMKN-45#1) for further experimental studies due to their strong inhibitory effects. To directly observe the change of protein of *DIMT1* in various tumor cell lines, we used the indirect immunofluorescence assay. Signals representing *DIMT1* staining were strongly decreased in the siMKN-45 cell line whereas they were significantly increased in the SGC-7901-D cells line (Figure 1H).

### Dimethyladenosine Transferase 1 Promotes Gastric Carcinoma Cell Proliferation In Vitro

**Figure 2.**
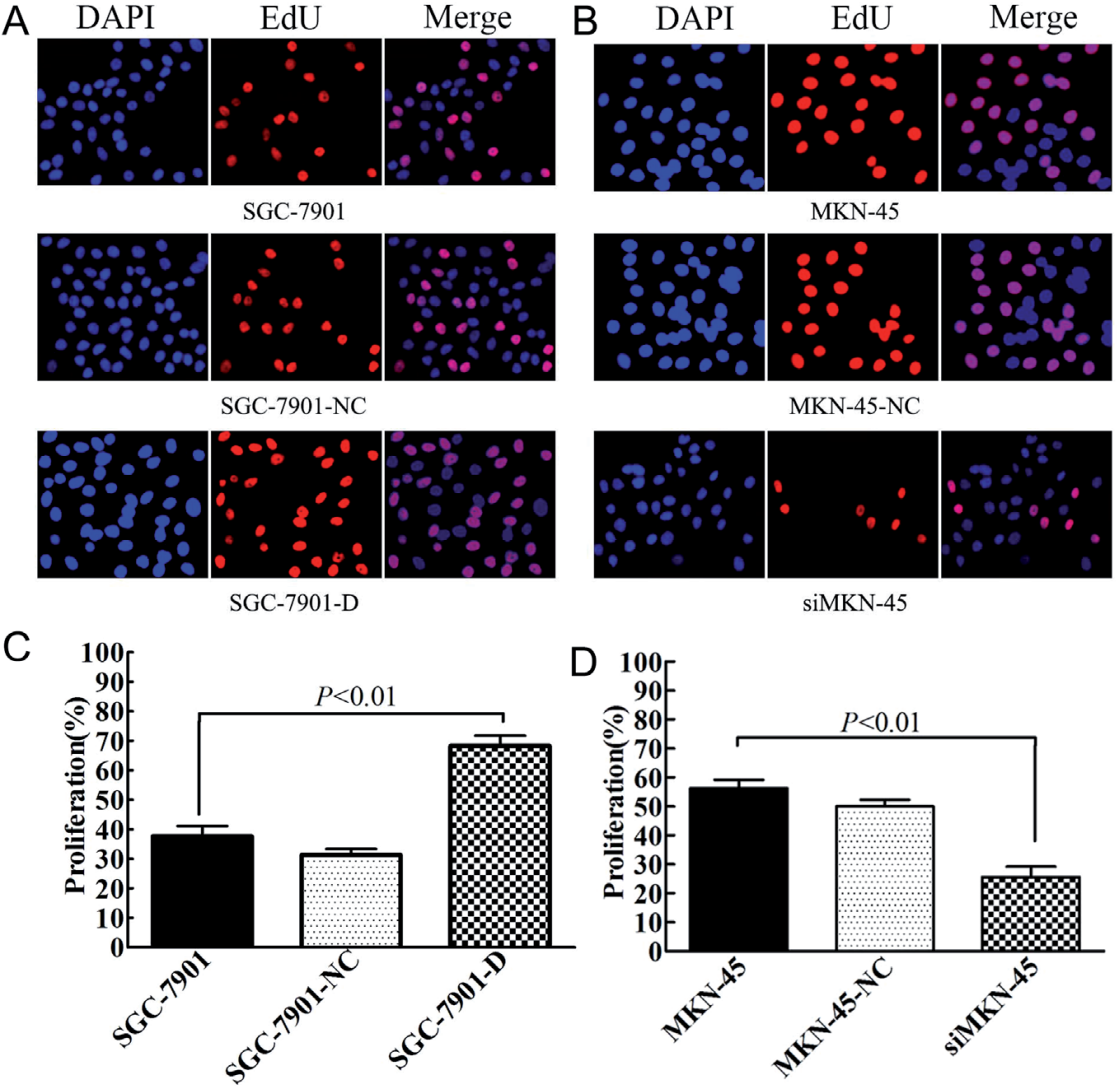
Dimethyladenosine transferase 1 (*DIMT1*) promoted gastric carcinoma cell proliferation *in vitro*. (A and C) Immunofluorescence staining of EdU indicated that the increased *DIMT1* expression promoted the proliferation activity in SGC-7901. (B and D) Decreased *DIMT1* expression inhibited the proliferation activity in MKN-45.

**Figure 3.**
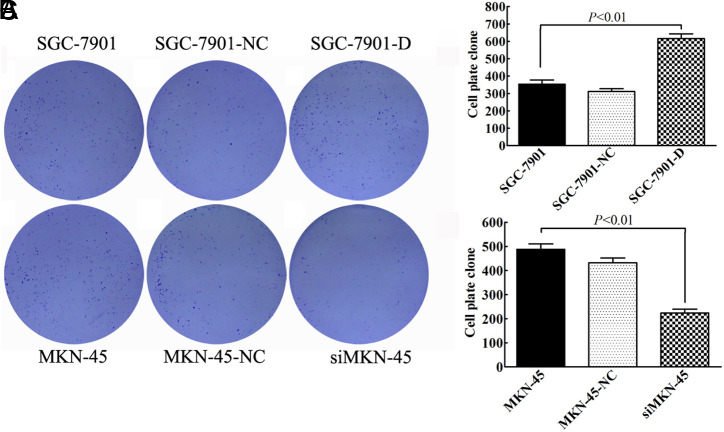
Dimethyladenosine transferase 1 (*DIMT1*) promoted gastric carcinoma cell growth *in vitro*. (A) Dimethyladenosine transferase 1 overexpression and knockdown cell variants were subjected to cell colony-formation assay. (B-C) The colonies are shown as histograms.

To study the effects of *DIMT1* on the proliferation of GC cells, we first determined the cell growth using an EdU assay. Our result showed that the EdU staining of the SGC7901 cell line with *DIMT1* over-expression was remarkably increased compared with the 2 control groups (Figure 2A and 2C), while the fluorescence intensity was significantly reduced by *DIMT1* depletion in the MKN-45 cell line (Figure 2B and 2D). In addition, the results of colony-formation assay indicated that the SGC-7901-D cells increased the colony number by 2.14-fold and 1.76-fold compared to SGC-7901-NC cells and SGC-7901 cells, respectively (Figure 3A top row and 3B). Consistently, down-regulated the *DIMT1* expression was decreased the colony-formation capacity, and, compared with the MKN-45-NC cells and MKN-45 cells, the siMKN-45 cells produced many fewer colonies (about 61.8% and 72.5%, respectively) (Figure 3A bottom row and 3C). The above results suggested that *DIMT1* can promote the GC cell growth and enhance cell colony formation *in vitro*.

### Dimethyladenosine Transferase 1 Contributes to Resistance Against Apoptosis In Vitro

When cells undergo apoptosis, a phosphatidylserine residue on the plasma membrane flips to the outside and is specifically recognized by Annexin V, while the necrotic cells can be stained only with PI. The general rule is that early apoptotic cells have Annexin V^+^ and PI^–^, whereas late apoptotic cells have both Annexin V^+^ and PI^+^. As shown in Figure 3A and 3B, our results show that the total percentage of apoptotic cells (viable apoptotic cell, UR + LR) changed from 6.76% in SGC7901 cells to 8.81% and 3.03% in SGC7901-NC cells and SGC-7901-D cells for 36 hours. Ectopic *DIMT1* expression rendered a decrease in the proportion of SGC-7901 cell line apoptosis (6.76% vs. 3.03%, *P* < .01). Consistently,* DIMT1 *knockdown promoted apoptosis in MKN-45 cells. The percentage of apoptotic cells in siMKN-45 cells increased 2.8-fold compared with MKN-45 cells in 36 hours (Figure 3A bottom row and 3C, *P* < .01). Surprisingly, the apoptosis level of the SGC-7901 and MKN-45 cell lines were infected with empty virus were increased. Furthermore, we also studied the effect of *DIMT1* expression on apoptosis by assessing *Caspase-3* activation by indirect immunity fluorescence. It showed that ectopic *DIMT1* expression led to remarkable increase in the *Caspase-3* fluorescent intensity in SGC-7901 cells, whereas the inhibition of *DIMT1* expression in MKN-45 cells resulted in significantly less *Caspase-3* activation.

### Dimethyladenosine Transferase 1 Enhanced Gastric Carcinoma Growth in a Xenograft Model

**Figure 5.**
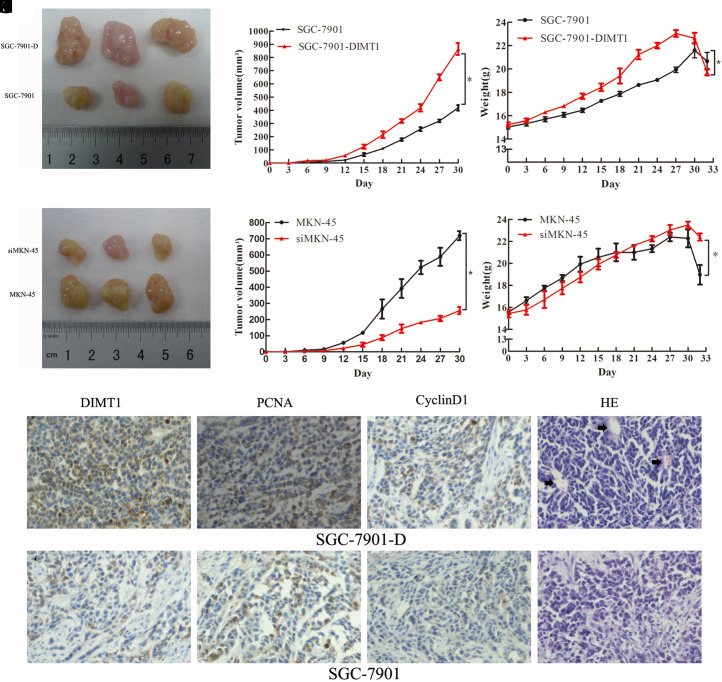
Dimethyladenosine transferase 1 (DIMT1) promoted gastric carcinoma cell proliferation *in vivo*. (A–C) In the overexpression group, the volume of xenograft tumors was enlarged manifestly, and the average overall weight was significantly increased (all * *P* < .01). (D–F) The knockout group had the opposite result (all * *P* < .01). (G–L) Immunohistochemistry for *DIMT1*, *PCNA*, and *CyclinD1* detection revealed that tumor cells in the overexpression group showed a higher positivity rate than that in the control group (magnification×200). (M and N) The newborn capillaries of xenograft tumors of the overexpression group more than the control group with hematoxylin and eosin-stained (magnification×200).

As anchorage-independent growth of carcinoma cells is a key feature of their tumorigenicity, we next evaluated the function of *DIMT1* on GC tumorigenesis *in vivo*. We subcutaneously injected a variety of GC cells into the flank regions of nude mice and monitored tumor growths after every 3 days. We found that tumor mass could be seen with the naked eye in the inoculated mice on day 6, with the tumor formation rate being 100%. Compared to the SGC-7901 group, the xenografts of the SGC-7901-D group were gained from day 15 post-treatment onwards, and with a ~2.5-fold increase in the final tumor volume (30 days, Figure 5A and 5B). The net weight of the nude mice of the overexpression group also showed a statistical difference during the beginning 21 days (*P* < .01, Figure 5C); interestingly, the difference had vanished on the 30th day (*P* > .01, Figure 5C). Meanwhile, there was a dramatic ~3.3-fold decrease in the average xenograft volume in mice that were inoculated with MKN-45 cells that had deleted *DIMT1* expression (*P* *< *.01, Figure 5D–F), and the net weight did not show a significant difference except on the 30th day between the subgroups.

Next, for each xenograft tumor, 5 random fields per slide were selected for H&E staining and IHC. Consistent with these data, the expression of *DIMT1*, *PCNA*, and *CyclinD1* proteins in the SGC-7901-D group of xenograft was higher than that of the SGC-7901 group (Figure 5G–L). Additionally, we also found that the newborn capillaries increased significantly with H&E-stained in the SGC-7901-D group (Figure 5M). These data indicated that* DIMT1 *has the potential to promote GC tumorigenicity *in vivo*. Otherwise, *DIMT1* knockdown could suppress carcinoma tumorigenesis.

### Dimethyladenosine Transferase 1 Activates PI3K/AKT Signaling Pathway and Induces PI3K/AKT-Regulated Gene Products

**Figure 6.**
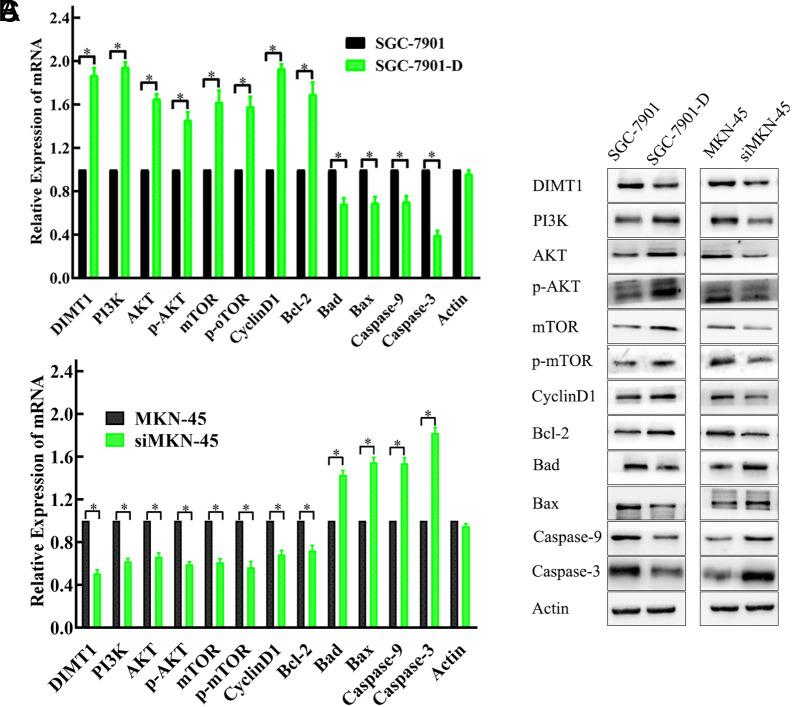
Dimethyladenosine transferase 1 (*DIMT1*) promoted proliferation and inhibited apoptosis via the activation of the *PI3K/AKT* pathway. The effects of *DIMT1* on *PI3K/AKT*-regulated cell proliferation and apoptosis-related genes. Expression levels of *DIMT1, PI3K, AKT, mTOR*, *p-AKT, p-mTOR, CyclinD1, Bcl-2, Bad, Bax, Caspase-3, Caspase-9*, and *Actin* were determined by quantitative RT-PCR (A and B, * *P* < .01) and Western blot (C).

We next attempted to elucidate the signaling pathways involved in the proliferation and apoptosis *in vitro* by *DIMT1*. Based on the changes in the biological characteristics of the cells, we speculated that *DIMT1* mutations could overactivate proliferation-related signal transduction pathways and lead to the uncontrolled growth of a tumor; meanwhile, it can adjust the expression of apoptosis-related genes. *PI3K/AKT* is one of the most common signaling pathways involved in regulating cell proliferation and apoptosis.^16-18^ Hence, we first assessed whether *PI3K/AKT *signaling pathway is involved in this process. As shown in Figure 6A and 6B, *PI3K*, *AKT*,* mTOR, p-AKT*,* p-mTORCyclinD1*, and *Bcl-2* mRNA were significantly decreased in *DIMT1*-silenced MKN-45 cells, and *Bad*,* Bax*, *Caspase-3*, and *Caspase-9* were significantly elevated; however, the decline range is more obvious. This protein expression was also noticeable accordingly (Figure 6C). Conversely, *DIMT1* overexpression in SGC-7901 cells prominently increased *PI3K*,* AKT, mTOR, p-AKT*,* p-mTOR*,* Cyclin D1*, and *Bcl-2* mRNA, and the protein levels were accompanied by decreased *Bad, Bax*, *Caspase-3*, and *Caspase-9* (Figure 6C and 6D).

From the above series of experiments, we have concluded that *DIMT1* depletion resulted in the repression of pro-survival genes, such as *AKT*, *CyclinD1*, and *Bcl-2,* while it induced the expression of the pro-apoptotic *Bad*, *Bax*, *Caspase-3*, and* Caspase-9.* Whereas, the overexpression of *DIMT1* led to the opposite changes in the expression of those mRNA and proteins. It means that the pro-growth and pro-survival functions of *DIMT1* in GC cells are partly mediated by the *PI3K/AKT* pathway activation.

## Discussion

Gastric carcinoma remains one of the most malignant carcinomas.^19^ Lack of standard therapy, rapid progress, poor prognosis, and high mortality are its typical characteristics. Therefore, it is very important to change its biologic behavior and ascertain potential novel therapeutic approaches. A previous study reported that *DIMT1*, a new member of the methyltransferase superfamily, may play specific roles in tumorigenesis.^9^ However, its biological role and the underlying molecular mechanisms contributing to the stimulative effects of the *DIMT1* on human GC cells are not elucidated well yet. Our previous study identified that *DIMT1* was highly expressed in GC tissues, and the high expression of *DIMT1* is closely correlated with differentiation and clinical TNM staging of GC, and it has a negative relation to the prognosis of the GC patients.^10^ These results suggested that *DIMT1* might be involved in GC progression regulation, and so we considered it could promote GC cell proliferation and anti-apoptotic. Based on our previous study, we conducted this deep and broad study. The effect of *DIMT1* on GC cell proliferation and apoptosis was investigated both *in vitro *and *in vivo.* We demonstrated for the first time that *DIMT1* could effectively promote GC cell proliferation and induce apoptosis by related researches. We thus hypothesize that *DIMT1* may be a potential tumor gene.

During culture, the growth of the siMKN-45 cells was significantly slower than that of MKN-45 cells. With other conditions remaining unchanged, MKN-45 cells spread to cover the bottom of the culture bottle which requires 2 days, while siMKN-45 cells need at least need 4 days or even longer. Whereas the SGC-7901-D cells were precisely the opposite, they grew very rapidly and sometimes were out of control. The time taken by these cells to cover the culture bottle is just only one-third to one-half of SGC-7901 cells. In addition, the results of plate formation assays were all similar. As the core gene of cell proliferation, *CyclinD1*, a cyclin that is crucial to the commitment to DNA synthesis,^20^ and *PCNA*, which is closely associated with cell DNA synthesis and plays an important role in tumor cell proliferation,^21^ were significantly upregulated in the SGC-7901-D cells. These results indicate that there was a close relationship between *DIMT1* and tumor cell proliferation and differentiation. In addition, we observed that these siMKN-45 cells were aggregated and closely connected and interdependent on each other under the microscope. Meanwhile, these cells were closely attached to the bottle wall and not easily digested by trypsin. More interestingly, electron microscopy images obtained at high magnification showed that the siMKN-45 cell shape has changed from pebble shape into polygonal or irregular or round or spindle shape. Furthermore, these aggregated cells grew slowly subtle protrusions, which can be entangled together like the human arms. A previous study shows that these protrusions can inhibit tumor cell migration, while these cells with smooth surface are strongly capable of migration.^22^ *DIMT1* knockout in MKN-45 cells was responsible for this phenomenon. But the phenomenon of the SGC-7901-D cells changed to round was not observed. We inferred that suppressed the expression of the *DIMT1* gene slow down the growth speed of cells and gradually decreases its migration ability. These results can provide a new research idea for invasion and metastasis of tumor cells.

It is well known that tumorigenesis is due to the disruption of the balance between cell proliferation and apoptosis.^23^ Generally, proliferation and apoptosis have always been considered to be a counter-balance paradox.^24,25^ The mRNA and protein results showed that pro-apoptosis genes *Bad*, *Bax*, *Caspase-3*, and *Caspase-9 *were upregulated, and the anti-apoptosis gene *Bcl-2* was downregulated in MKN-45 cells after* DIMT1* knockout. Indeed, the apoptotic cell numbers of siMKN-45 cells should increase significantly compared with MKN-45 cells. Contrarily, the results of flow cytometry were not as so significant as we envisioned. Similar trends were found in SGC-7901-D cells, apoptosis-related factors and proteins were change significantly, but the reduction in numbers of apoptotic cells was not significant. The apoptosis level of tumor cells is also low. The reduction in apoptosis cells may not be evident after overexpression or amplification of oncogenes. Theoretically, the apoptosis level should increase significantly after the oncogene was knocked out. A repeat experiment has produced similar results. With the deep analysis of these results, we think that after the *DIMT1 *knockdown, tumor cellular will initiate a self-protection mode. The ability of anti-apoptosis was passive activation, and the decrease in the level of apoptosis is not apparent. Once the genes associated with proliferation were activated, they might have a cascade effect, and proliferation ability enlarged continuously. Through relevant research, we identified the role of *DIMT1* in promoting GC cell growth and inducing apoptosis. Although the latter was not as remarkable as expected, it occurs. This gives us a revelation that suppressing tumor growth is a more effective strategy for anti-tumor therapies.

In animal experiments, we found that transplanted tumors in both the knockdown and overexpression groups grew slowly. There is no difference between tumor sizes in nude mice within 15 days after inoculation.^26^ We think that this is largely a consequence of tumor microenvironment change because these cells take a long time to adapt to the environment. After 15 days, we observed a remarkable acceleration in the growth of the nude mice tumor transplantation model, especially in the overexpression group. However, the difference started to be visible in the 2 experimental groups, and the results of the experiment are in good agreement with our hypothesis. There was a large discrepancy in changes in body weight between expected results and actual results, and the weight of each group did not show any significant difference throughout the follow-up time. By analyzing, we find that it has to do with the tumor body: the transplanted tumor grew fast and very large in the overexpression model. These animals were emaciation and malnutrition. Growth of the subcutaneous transplanted tumors decreased, and tumor mass was smaller in the knockout group, and the mice were in good condition. Nevertheless, significant changes in net weight were observed, and the net weight had significant differences in each group. It is difficult to found the net weight of the nude mouse; therefore, the results had low precision in the process of growth. We believed that the evaluation of tumor cell proliferation ability is the most scientific method to compare the transplanted tumor size over the same period. So, the results were objective, factual, scientific, and rational.

Multiple pathways are involved in tumor cell proliferation, differentiation, and apoptosis, including the *PI3K/AKT* pathway, *Hh *signaling pathway, nuclear factor-κB signaling pathway, and so on.^27-29^ This study indicated that *DIMT1* promotes cancer cell proliferation and suppresses apoptosis via the *PI3K/AKT* pathway activation. *PI3K* is a member of the intracellular lipid kinase family, which catalyzes the generation of *PIP3* from *PIP2*.^30^ Activated *AKT* translocates to the nucleus and activates *mTOR *and downstream targets.^31,32^ Our finding supported the fact that SGC-7901-D cells activate the *PI3K/AKT* pathway, while *DIMT1* knockout silencing reduces the* PI3K/AKT* phosphorylation. Meanwhile, we also found that inhibiting the *PI3K/AKT* signaling pathway may be possible for treating gastric carcinoma. These results identified *DIMT1* as a potential enhancer of proliferation potential in gastric carcinoma. In our future studies, we will continue to elucidate whether *DIMT1* impacts molecules from other pathways and elucidate its effects on human GC’s biological behaviors and their underlying mechanism.

It has been found in subcutaneous tumor-forming experiments in nude mice that upregulation of *DIMT1 *can promote tumor neovascularization. The relevant mechanism of *DIMT1* on tumor neovascularization is not clear at present, and we will study the mechanism of related phenomena in the future. The overall study of *DIMT1* in the field of gastric cancer is not in depth. Next, we will conduct comprehensive studies combining miRNA, long non-coding RNA, and circRNA.

## Conclusion

We demonstrated that *DIMT1* plays a crucial role in growth inductive and apoptosis inhibitory. The *PI3K/AKT* signaling pathway and its regulated gene products are activated in *DIMT1*-overexpressing cells. Dimethyladenosine transferase 1 plays a significant part in promoting GC cell growth and providing apoptosis resistance; therefore, it offers a promising target to treat and prevent gastric carcinoma.

## Figures and Tables

**Figure 4.**
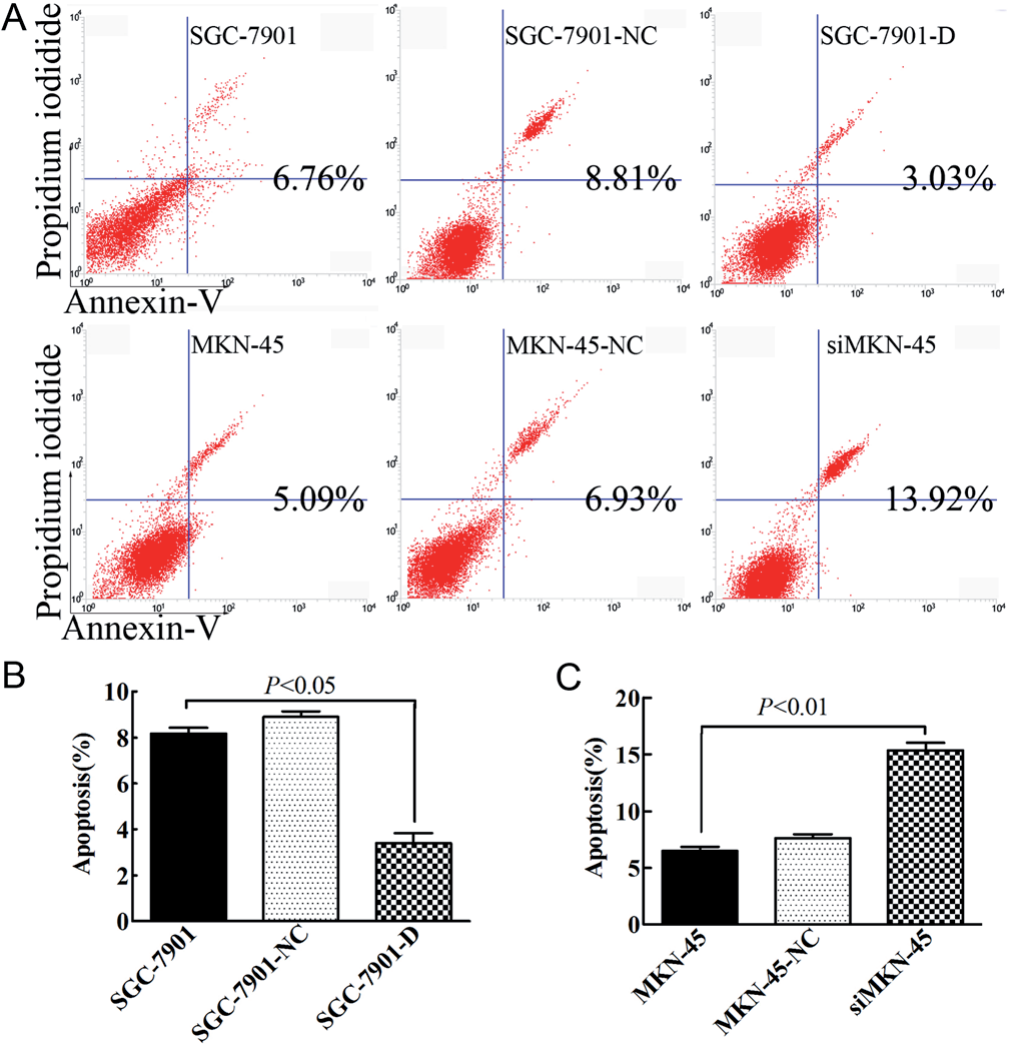
Dimethyladenosine transferase 1 (*DIMT1*) contributes to resistance apoptosis *in vitro*. (A) Dimethyladenosine transferase 1 overexpression and knockdown cell variants were subjected to cell apoptosis analysis using an Annexin V/propidium iodide assay. (B and C) The percentages of apoptotic cells are shown as histograms.
